# Oxidized Albumin and Cartilage Acidic Protein-1 as Blood Biomarkers to Predict Ischemic Stroke Outcomes

**DOI:** 10.3389/fneur.2021.686555

**Published:** 2021-11-30

**Authors:** Takahiro Kuwashiro, Kazuhiro Tanabe, Chihiro Hayashi, Tadataka Mizoguchi, Kota Mori, Juro Jinnouchi, Masahiro Yasaka, Yasushi Okada

**Affiliations:** ^1^Department of Cerebrovascular Medicine and Neurology, Clinical Research Institute, National Hospital Organization, Kyushu Medical Center, Fukuoka, Japan; ^2^Medical Solution Promotion Department, Medical Solution Segment, LSI Medience Corporation, Tokyo, Japan; ^3^Kyushu Pro Search Limited Liability Partnership, Fukuoka, Japan

**Keywords:** acute ischemic stroke, cartilage acidic protein-1, oxidative stress, oxidized albumin, mass spectrometry, LC-MS/MS, biomarker (BM)

## Abstract

**Background:** There is high demand for blood biomarkers that reflect the therapeutic response or predict the outcomes of patients with acute ischemic stroke (AIS); however, few biomarkers have been evidentially verified to date. This study evaluated two proteins, oxidized albumin (OxHSA) and cartilage acidic protein-1 (CRTAC1), as potential prognostic markers of AIS.

**Methods:** The ratio of OxHSA to normal albumin (%OxHSA) and the level of CRTAC1 in the sera of 74 AIS patients were analyzed on admission (day 0), and at 1 and 7 days after admission. AIS patients were divided into two groups according to their modified Rankin Scale (mRS) at 3 months after discharge: the low-mRS (mRS < 2) group included 48 patients and the high-mRS (mRS ≥ 2) group included 26 patients. The differences in %OxHSA and CRTAC1 between the two groups on days 0, 1, and 7 were evaluated.

**Results:** The mean %OxHSA values of the high-mRS group on days 0, 1, and 7 were significantly higher than those of the low-mRS group (*p* < 0.05). The CRTAC1 levels continuously increased from day 0 to day 7, and those of the high-mRS group were significantly higher than those of the low-mRS group on day 7 (*p* < 0.05).

**Conclusions:** These results suggest that higher %OxHSA and CRTAC1 are associated with poor outcomes in AIS patients. An index that combines %OxHSA and CRTAC1 can accurately predict the outcomes of AIS patients.

## Introduction

Acute ischemic stroke (AIS) caused by a thrombus is one of the most lethal and physical disabling cerebrovascular diseases (1). To minimize cellular necrosis and improve the outcomes of AIS patients, preventing further generation of reactive oxygen species (ROS) in the penumbra is an important issue (2). Edaravone, a free radical scavenger, plays an important role in removing ROS radicals and reduces the risk of further destruction of neuronal networks (3). However, administration of too much edaravone causes serious renal failure, so the administered dose needs to be strictly controlled. We hypothesized that if the redox state of AIS patients could be monitored correctly, we could provide optimal, individualized doses of radical scavengers, which should improve patient outcomes.

Several studies have investigated the relationship between the outcomes of AIS patients and oxidative stress, aiming to verify biomarkers that can predict the outcomes using non-invasively obtained specimens, such as urine or blood (4–11). However, the experimental evidence regarding oxidative stress in humans remains lacking, mainly because it has not been sufficiently verified whether these biomarkers reflect actual oxidative stress (12).

Human serum albumin includes 17 disulfides and one unpaired thiol residue (Cys 34), and this unpaired thiol generates a disulfide bond with a free cysteine in plasma during periods of increased oxidative stress, yielding oxidized albumin (OxHSA) (13). Although the ratio of the oxidized form to the reduced form in healthy individuals (%OxHSA) is <40%, it increases to more than 50% in patients with conditions that expose them to high levels of oxidative stress, e.g., diabetes (14, 15), cardiovascular disease (16), Parkinson's disease (17), or liver cirrhosis (18). Since albumin is a major protein in plasma (3.5–5.5 g/dL), %OxHSA is considered to be the most reliable indicator of the whole-body redox state. Some studies have evaluated %OxHSA as an AIS biomarker. Moon et al. revealed that %OxHSA was elevated in the cerebrospinal fluid of AIS patients (19).

Recent studies have revealed that not only reducing oxidative stress, but also restoring nervous system function in the penumbra affects the outcomes of AIS patients. AIS patients often exhibit continued functional recovery for many years after their initial injury (20), which has also been observed in animal stroke models (21–23). Cartilage acidic protein-1 (CRTAC1) is a plasma protein that binds to the Nogo receptor-1 (NgR1) in the brain (24–26). Nogo inhibits neural regeneration, so binding of CRTAC1 to NgR1 blocks the interaction between the receptor and Nogo, thereby promoting axon growth. Takase et al. revealed that the CRTAC1 (LOTUS) in blood contributes to promote nerve regeneration in mice overexpressing CRTAC1 (26). We hypothesized that the plasma level of CRTAC1 changes depending on the degree of AIS, and may affect AIS patient outcomes. Although there are currently no medications for regenerating the nervous system, plasma levels of CRTAC1 may provide scientists clues toward developing new medications or treatments for AIS.

In this study, we aimed to evaluate two biomarkers, OxHSA and CRTAC1, as potential prognostic biomarkers in AIS patients.

## Materials and Methods

### Participants

This study prospectively recruited patients with AIS from March 2017 to February 2018, to search for potential prognostic biomarkers. Institutional Review Board (IRB) approval was obtained from both the National Hospital Organization Kyushu Medical Center (IRB registration number, 16C132) and LSI Medience Corporation (MS/Shimura 16-22) for use of the patients' clinical information and plasma samples.

### Diagnosis of AIS

Consecutive patients (*n* = 74) were enrolled who were admitted to the National Hospital Organization Kyushu Medical Center within 24 h after the symptoms of AIS were recognized. The number of participants was calculated by the estimated difference of %OxHSA between two groups (poor and mild outcomes, 2%), alpha-error (0.05), beta-error (0.2), and the standard deviation of %OxHSA obtained from validation (2.7%). AIS diagnosed by brain imaging, including computed tomography (CT) and magnetic resonance imaging (MRI), in all patients was classified as atherothrombotic brain (ATBI), cardioembolic (CE) or lacunar (LAC) infarction, and unclassified (UC) based upon the diagnostic criteria of the Classification of Cerebrovascular Disease III proposed by the National Institute of Neurological Disorders and Stroke (27), and of the Trial of Org 10172 in Acute Stroke Treatment (TOAST) study (28). Patients with serious renal, liver, respiratory, or cardiac conditions, and patients with infectious diseases, Parkinson's disease, schizophrenia, severe dementia, or severe cognitive impairment (mRS ≥ 4) at stroke onset were excluded from the present study. Informed consent was obtained from all patients who participated in the study. All patients underwent full physical and neurological examinations to obtain NIH stroke scale and mRS scores on admission and, at discharge. The mRS score was obtained again at the 3-month follow-up. Vascular risk factors, medical history, and smoking status were also recorded. Laboratory blood tests related to liver functions, lipid metabolism, and renal function were performed on admission. CRTAC1 and %OxHSA were analyzed at days 0 (on admission), 1, 7, and 14; however, data from day 14 were excluded because more than 40% of the patients were discharged from the hospital before then.

### Blood Sampling for %OxHSA and CRTAC1

Albumin in plasma rapidly binds to free cysteine and generates OxHSA after blood sampling, so preventing further oxidation of albumin is crucial. Kubota et al. discovered that addition of an acid (0.5 M citrate buffer, pH 4.2) to plasma immediately after blood sampling prevented further oxidation (29). To avoid the need for adding citrate buffer to separated plasma, thereby enhancing the ease of use of the test, we developed a new vacuum blood collection tube containing 0.5 mol/L citrate buffer (pH 4.2) in cooperation with NIPRO Corporation (Osaka, Japan). Approximately 2 mL of whole blood was collected from AIS patients using this vacuum sampling tube. Each blood sample was centrifuged (2000 g, 4°C for 15 min,), and the plasma was either transferred to a 1-mL microtube within 4 h after blood sampling, or the sampling tubes with blood cells were stored at 4°C and the plasma separated within 24 h. Separated plasma was stored at −80°C and %OxHSA was analyzed within 3 months.

### %OxHSA Analysis

Plasma samples (25 μL) were diluted with 1 mL of 50 mM phosphate buffer (pH 6.0). The diluted sample was subsequently applied to a Bond Elute C18 EWP column, followed by washing with 1 mL of solvent A (0.1% formic acid, 9.9% acetonitrile and 90% water [%v/v]). Albumin was then eluted using 1 mL of solvent B (0.1% formic acid, 9.9% water and 90% acetonitrile [%v/v]). Liquid chromatography-mass spectrometry (LC-MS) data were acquired using a liquid chromatography system (Agilent HP1200, Agilent Technologies, Palo Alto, CA) coupled with an electrospray ionization quadrupole time-of-flight mass spectrometer (Agilent 6520, Agilent Technologies). The liquid chromatograph and mass spectrometer were linked with a stainless steel microtube (0.1-mm internal diameter, 1-m length), and no column was used. The mobile phase was 0.1% formic acid and 40% acetonitrile in water (%v/v), and albumin was eluted at a flow rate of 50 μL/min at room temperature under isocratic conditions. The mass spectrometer was operated in the positive mode with a capillary voltage of 4000 V. The nebulizing gas pressure was 20 psi and the dry gas flow was 5 L/min at 325°C. The mass range was set from m/z 800 to 3000. The injection volume was 2 μL, and the total measurement time was 3.0 min. The LC-MS raw spectrum was converted to CSV format using Mass Hunter Export (Agilent Technologies) and deconvoluted using Excel VBA (Excel 2010, Microsoft, Redmond, WA) software.

### CRTAC1 Analysis

The plasma concentration of CRTAC1 was analyzed using a human CRTAC1 ELISA kit (CUSABIO, Houston, TX) according to the manufacturer's instructions.

### Principal Component Analysis

Principal component analysis (PCA) was performed for CRTAC1 (days 0, 1, and 7), %OxHSA (days 0, 1, and 7), and laboratory biochemistry tests on admission using SIMCA software (version 13.0.3; Umetrics, Umeå, Sweden). The main purpose for conducting PCA was to condense the large amount of variable information into a smaller set of new composite dimensions with a minimum loss of information, and to discover underlying characteristics or relationships in the large dataset.

### Statistical Analysis

The mRS-predictive index (mRS-PI) were obtained by the following procedure. First, the values of d-dimer, %OxHSA and CRTAC1 are preliminarily normalized by unit variance and zero mean centering, then the coefficients for the equation were optimized by sequential quadratic programming provided by Excel Solver to minimize *p*-value of student *t*-test between two groups.

## Results

### Demographic Characteristics

The 74 AIS patients were divided into two groups based on their mRS score at 3 months after discharge. The low-mRS group included 48 patients with an mRS score of 0 or 1, and the high-mRS group included 26 patients with an mRS score ≥2. The cutoff was determined according to whether the patients had impairments. The characteristics of the patients are summarized in Table 1.

**Table 1:** Characteristics of the patients.

**Features**		**mRS <2** **(***n*** = 48)**	**mRS ≥ 2** **(***n*** = 26)**	***p*** **-value**
Age (years)		70.4 (±14.2)	74.2 (±10.7)	0.20
Gender	Male (%)	32 (66.7%)	13 (50.0%)	
Stroke subtypes	ATBI	5	3	
	LAC	11	4	
	CE	8	7	
	UC	24	12	
mRS 3-months	0	26	-	
post-discharge	1	22	-	
	2	-	6	
	3	-	10	
	4	-	9	
	>5	-	1	
NIHSS	Admission	1.46 (±1.47)	4.92 (±4.74)	0.0011
Missing data	OxHSA	2	0	
	CRTAC1	3	0	
	Laboratory test	4	0	

*OxHSA, oxidized albumin; CRTAC1, cartilage acidic protein-1; ATBI, atherothrombotic brain infarction; LAC, lacunar infarction; CE, cardioembolic infarction; UC, unclassified infarction*.

### Validating the New Vacuum Blood Collection Tube for %OxHSA

We obtained blood samples using the newly developed tube, and then left the collected samples at room temperature for 2 and 4 h, or at 4°C for 1, 3, and 10 days, without separating blood cells. We also stored the separated plasma at −20°C or −80°C for 1 and 3 months. We compared the %OxHSA of those samples to that of the plasma that was immediately separated after blood sampling. As a result. The validation tests demonstrated that %OxHSA was stable (relative error ≤ 5%) when the collection tube containing blood was left at room temperature for up to 4 h, or at 4°C for up to 10 days. After plasma separation, %OxHSA was stable at −80°C for 3 months; however, it was not stable at −20°C for ≤ 1 month (Supplementary Table I). Considering these results, we ensured that each blood sample was centrifuged and the plasma transferred to a microtube within 4 h after blood sampling. Alternatively, blood sampling tubes containing blood cells were stored in a refrigerator at 4°C and the plasma was separated within 24 h. Separated plasma was stored at −80°C and %OxHSA was analyzed within 3 months. We further evaluated the precision, accuracy, and robustness of the mass spectrometry analysis (Supplementary Table II). Both within-day and between-day reproducibility, and dilution, freeze–thaw, and short-period stability were acceptable (relative error and coefficient of variance % ≤ 5%).

### Relationship Between Outcome of AIS Patients and Laboratory Test Results on Admission

Table 2 shows the means and the standard deviations of the laboratory test results related to vascular risk (blood pressure), liver function (AST, ALT, LDH, ALP, γ-GTP, T-Bil, platelets, albumin, and PT-INR), lipid metabolism (total cholesterol, LDL-cholesterol, HDL-cholesterol, and triglycerides), sugar metabolism (BS, FBS, and HbA1c), and renal function (BUN, Cr, urinary acid, and eGFR) on admission. Comparisons of the low- and high-mRS groups revealed no significant differences in these values between the two groups (Student's *t*-test, *p* > 0.1). Only d-dimer was markedly elevated in the high-mRS group (*p* < 0.05) compared to the low-mRS group.

**Table 2:** Laboratory test results on admission for the low-mRS and high-mRS score groups.

**Biomarker**	**mRS <2** **(***n*** = 48)**	**mRS ≥ 2** **(***n*** = 26)**	***p*** **-value**
	**Ave. (St. Dev.)**	**Ave. (St. Dev.)**	
Systolic arterial pressure (mmHg)	154.8 (±26.6)	160.3 (±29.2)	0.767
Diastolic pressure (mmHg)	84.2 (±15.6)	88.8 (±19.3)	0.807
White blood cells	6617 (±1805)	7308 (±1919)	0.264
Red blood cells	461.1 (±72.1)	461.5 (±85.8)	0.891
Hematocrit (vol%)	40.3 (±7.8)	41.6 (±6.2)	0.735
Hemoglobin (g/dL)	14.2 (±2.1)	14.1 (±2.3)	0.818
Platelet (× 10^4^/μl)	22.5 (±7.7)	21.2 (±6.3)	0.702
AST (U/L)	25.4 (±8.4)	26.7 (±9.9)	0.429
ALT (U/L)	24.2 (±18.0)	23.3 (±18.2)	0.580
LDH (U/L)	228.8 (±58.5)	258.0 (±85.5)	0.399
ALP (U/L)	249.9 (±70.1)	248.2 (±86.2)	0.957
γ-GTP (U/L)	49.5 (±42.3)	34.2 (±35.7)	0.554
T-Bil (mg/dL)	0.683 (±0.439)	0.738 (±0.381)	0.372
Total cholesterol (mg/dL)	198.7 (±54.0)	197.9 (±36.5)	0.414
LDL-cholesterol (mg/dL)	110.5 (±38.3)	112.3 (±31.2)	0.456
HDL-cholesterol (mg/dL)	49.7 (±11.0)	55.2 (±17.8)	0.743
Triglycerides (mg/dL)	185.9 (±267.5)	127.3 (±62.4)	0.272
TP (g/dL)	7.05 (±0.52)	7.03 (±0.56)	0.875
Albumin (g/dL)	4.13 (±0.34)	4.04 (±0.40)	0.176
CPK (U/L)	100.1 (±59.4)	95.2 (±51.2)	0.397
BUN (mg/dL)	16.8 (±5.3)	17.5 (±7.6)	0.855
Cr (mg/dL)	1.12 (±1.48)	0.85 (±0.33)	0.459
eGFR (ml/min/1.73 m^2^)	68.5 (±25.8)	66.5 (±23.0)	0.653
Urinary acid (mg/dL)	6.07 (±1.62)	5.62 (±1.29)	0.999
FBS (mg/dL)	113.9 (±54.4)	108.1 (±37.2)	0.832
BS (mg/dL)	149.0 (±112.0)	132.3 (±76.1)	0.656
HbA1c (%)	6.19 (±2.39)	5.79 (±1.70)	0.684
CRP (mg/dL)	0.26 (±0.25)	1.24 (±3.22)	0.123
PT-INR	1.08 (±0.24)	1.01 (±0.08)	0.101
APTT (seconds)	29.7 (±3.7)	29.1 (±2.6)	0.379
Fibrinogen (mg/dL)	307.0 (±65.3)	311.2 (±85.4)	0.829
D-dimer (μg/mL)	0.97 (±1.2)	3.30 (±4.99)	*0.027

*mRS, modified Rankin scale; AST, aspartate aminotransferase; ALT, alanine aminotransferase; LDH, lactate dehydrogenase; ALP, alkaline phosphatase; γ-GTP, gamma-glutamyltransferase; T-Bil, total bilirubin; LDL-cholesterol, low-density lipoprotein cholesterol; HDL-cholesterol, high-density lipoprotein cholesterol; TP, total protein; CPK, creatine phosphokinase; BUN, blood urea nitrogen; Cr, creatinine; eGFR, estimated glomerular filtration rate; FBS, fasting blood sugar; BS, blood sugar; HbA1c, hemoglobin A1c; CRP, C-reactive protein; PT-INR, prothrombin time-international normalized ratio; APTT, activated partial thromboplastin time*.

* *p < 0.05*.

### Change in %OxHSA and CRTAC1 Levels From Admission to Day 7

**Figure 1:**
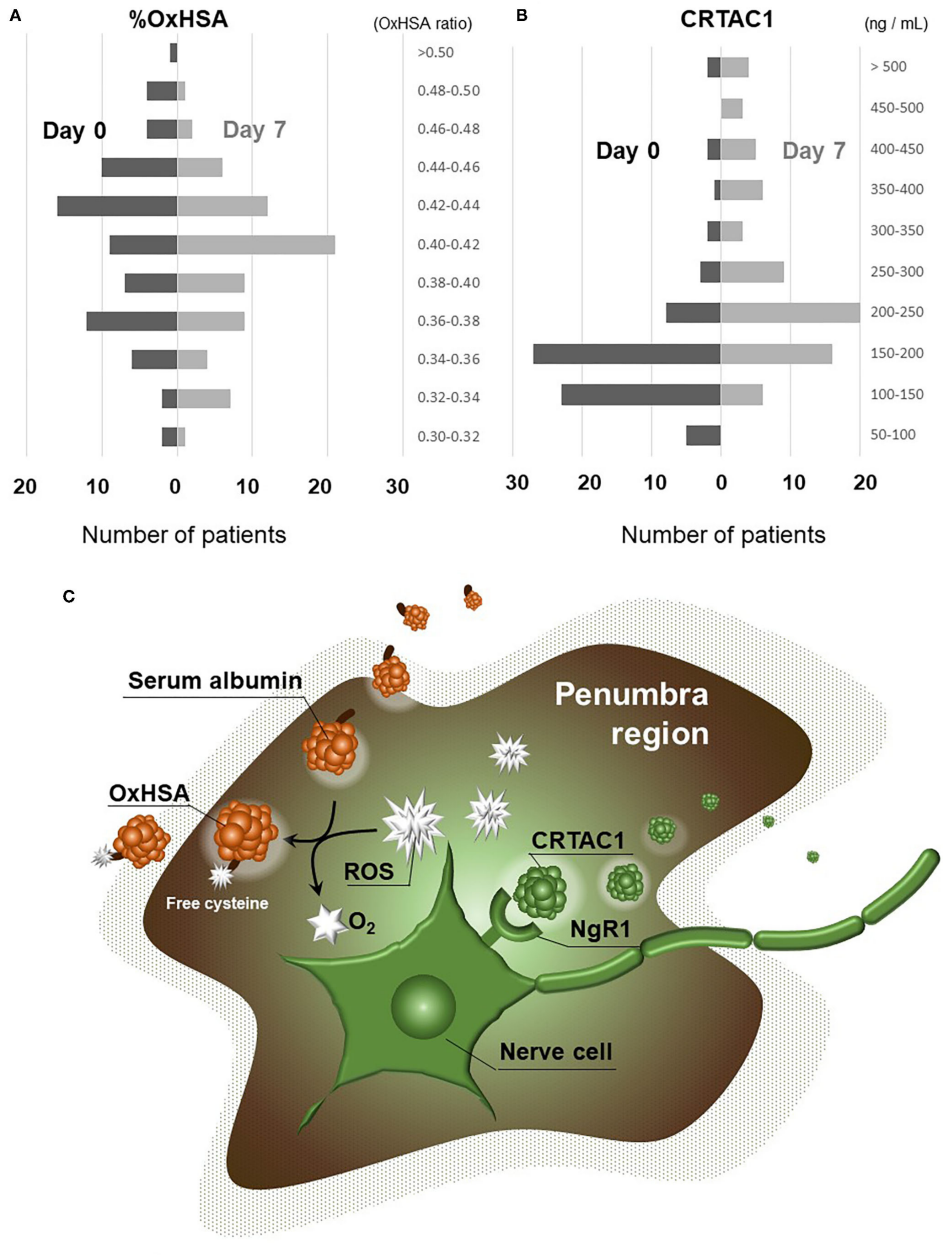
Changes in %OxHSA and CRTAC1 from day 0 to day 7. **(A)** Histogram showing the levels of %OxHSA classified into 0.02-unit (2%) increments is displayed on the left (day 0) with black bars, and on the right (day 7) with gray bars. **(B)** Histogram with levels of CRTAC1 classified into 50-ng/mL increments is displayed on the left (day 0) with black bars, and on the right (day 7) with gray bars. **(C)** An illustration of OxHSA and CRTAC1 functions in the penumbra. OxHSA, oxidized albumin; CRTAC1, cartilage acidic protein-1.

Figure 1 shows the changes in %OxHSA and CRTAC1 levels from admission to day 7. %OxHSA decreased in 48 (67%) of 72 patients (2 with missing data) over the 7-day period. The average %OxHSA on admission (41.1%) decreased slightly to 40.1% on day 7 (*p* = 0.15, Figure 1A). Supplementary Figure I shows the typical pattern of changes in %OxHSA during hospitalization for a 73-year-old man in the high-mRS group. On the other hand, increases in CRTAC1 were observed in 58 (82%) of 71 patients (3 with missing data), and the average CRTAC1 value on admission (187.5 ng/mL) was elevated significantly to 276.1 ng/mL on day 7 (*p* <10^−5^, Figure 1B). The putative functions of OxHSA and CRTAC1 are illustrated in Figure 1C. These results suggested that OxHSA increases with AIS and gradually decreases after commencement of treatment. On the other hand, CRTAC1 starts to increase after the onset of AIS, to restore nervous system function.

### Principal Component Analysis

**Figure 2:**
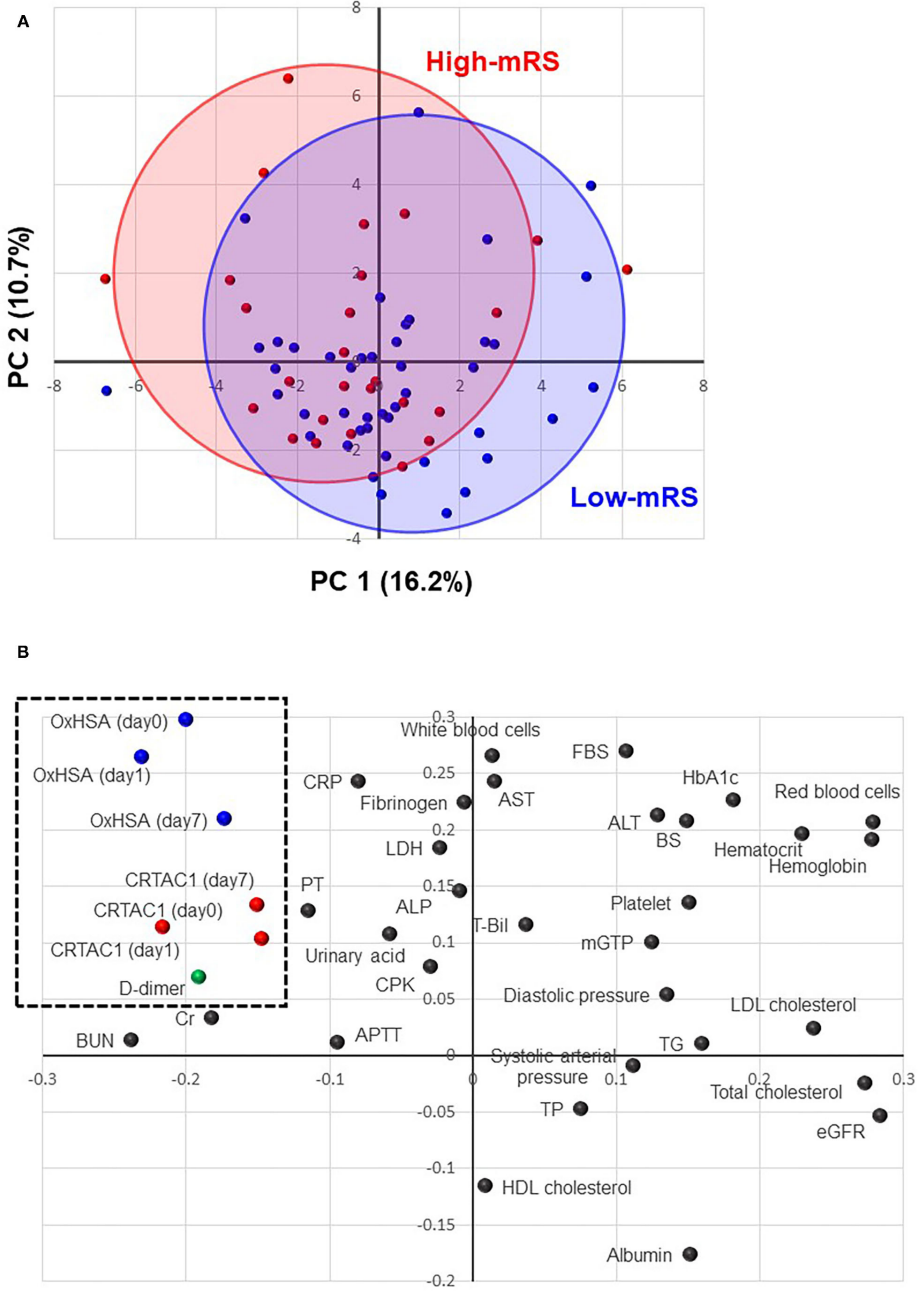
Principal component analysis (PCA). **(A)** A score plot of the first and second components of PCA analysis. Red solid circles indicate high-mRS patients, and the red oval shows the distribution of the high-mRS group. Blue solid circles indicate low-mRS patients, and the blue oval shows the distribution of the low-mRS group. **(B)** A loading plot of the first and second PCA components: each dot represents biomarkers, such as %OxHSA, CRTAC1, and laboratory test results. The biomarkers located in the upper-left corner (enclosed with a dotted-line square) are elevated in the high-mRS group. mRS, modified Rankin scale; OxHSA, oxidized albumin; CRTAC1, cartilage acidic protein-1.

PCA was performed to understand the distribution of patients in the low- and high-mRS groups, the similarity and dissimilarity of biomarker expression patterns, and biomarker contributions to the mRS score. Figure 2A shows the PCA score plot expressing the distribution of patients in both the low- and high-mRS groups. The distribution of patients in the high-mRS group (Figure 2A, red) was slightly shifted up and to the left relative to that of patients in the low-mRS group (Figure 2A, blue). Figure 2B shows the loading plot, which indicates the similarity and dissimilarity of the biomarker expression patterns. Red blood cells, hematocrit, and hemoglobin were located in an adjacent area in the upper-right corner, which suggests that the expression patterns of these indicators among AIS patients are relatively similar. The axes of the score and loading plots were interlocked to allow comparison of the two plots; thus, the biomarkers located in the upper-left side in the loading plot (Figure 2B) were elevated in the high-mRS group. CRTAC1, %OxHSA, and d-dimer were located in an adjacent area in the upper-left corner of the plot, which suggests that their expression patterns are similar, and that they tended to be increased in the high-mRS group. The complete score and loading information are described in Supplementary Tables III, IV.

### Significant Differences in CRTAC1 and %OxHSA Between Low- and High-MRS Groups

**Figure 3:**
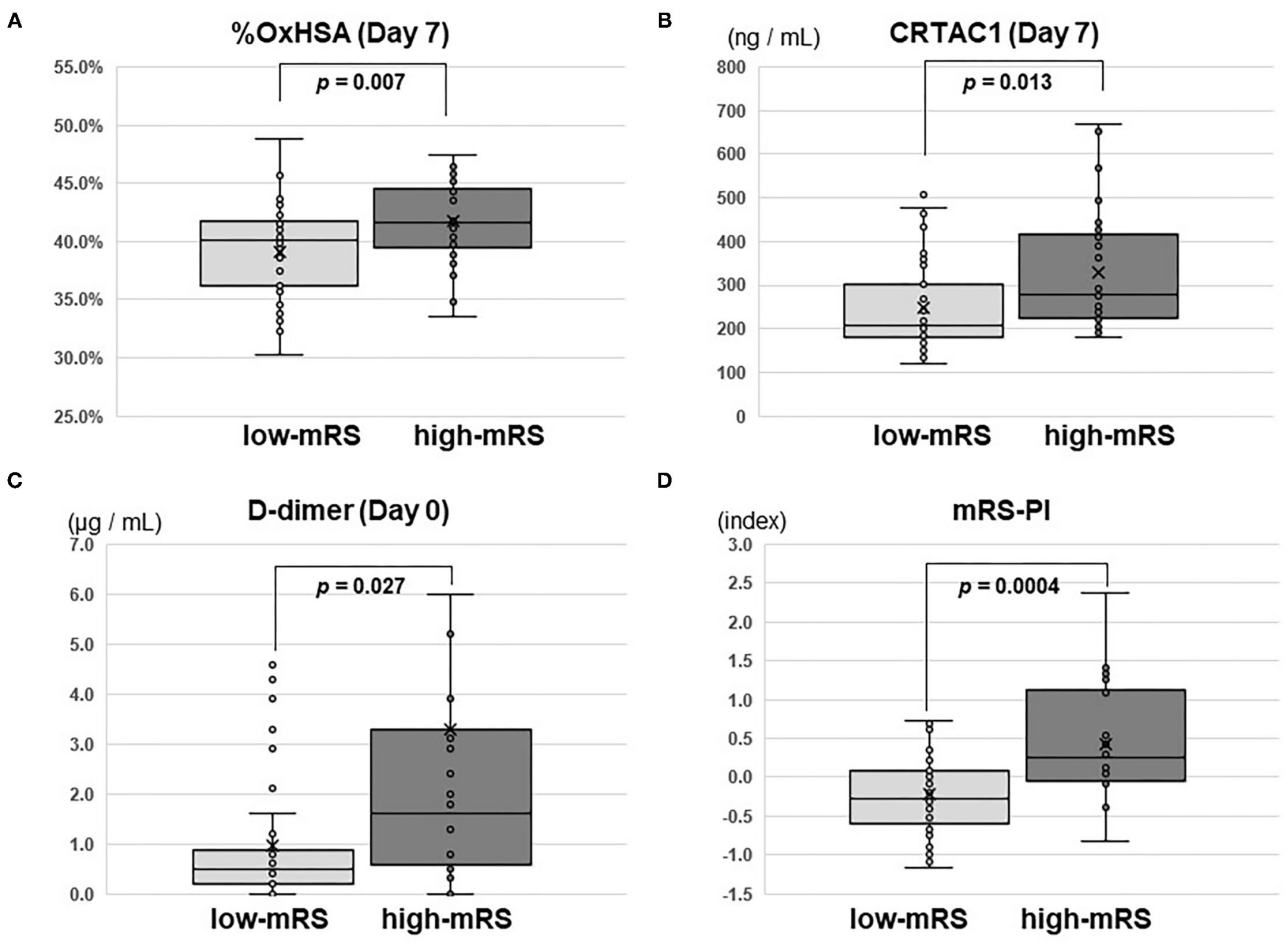
Box-whisker plots of %OxHSA, CRTAC1, D-dimer, and mRS-PI. Box-whisker plots of **(A)** %OxHSA (day 7), **(B)** CRTAC1 (day 7), **(C)** D-dimer (day 0), and **(D)** mRS-PI. Student's *t*-test *p*-values for comparisons between low-mRS and high-mRS groups are shown. OxHSA, oxidized albumin; CRTAC1, cartilage acidic protein-1; mRS, modified Rankin square; PI, predictive index.

Comparisons of CRTAC1 and %OxHSA on days 0, 1, and 7 between the low-mRS and high-mRS groups revealed that %OxHSA in the high-mRS group was significantly elevated at each time point compared to that of the low-mRS group (Table 3; Figure 3A). The CRTAC1 level in the high-mRS group was markedly increased compared to that of the low-mRS group, only on day 7 (Table 3; Figure 3B). As shown in Table 2, the level of d-dimer in the high-mRS group was also increased compared to that in the low-mRS group on admission (Figure 3C). The correlations between %OxHSA, CRTAC1, and d-dimer were positive but not strong (*r* = 0.25, 0.12, and 0.22 between d-dimer and %OxHSA, %OxHSA and CRTAC1, and CRTAC1 and d-dimer, respectively), i.e., the relationships between these three markers were complementary (Supplementary Figure II). The mRS-predictive index (mRS-PI), defined by the following equation, showed a significant increase in the high-mRS group (*p* = 0.0004, Figure 3D):


$$\begin{array}{l} mRS-PI=0.23\times (d-dimer)+0.44\times (\%OxHSA)\\ \;+0.33\times (CRTAC1), \end{array}$$

where the values of d-dimer, %OxHSA and CRTAC1 are preliminarily normalized by unit variance and zero mean centering. The coefficients for this equation were optimized by sequential quadratic programming, an iterative method for constrained non-linear optimization, provided by Excel Solver.

**Table 3:** %OxHSA and CRTAC1 levels in the low-mRS and high-mRS score groups over a 7-day period.

**Sampling**	**Biomarker**	**mRS <2 (***n*** = 48)**	**mRS ≥ 2 (***n*** = 26)**	***p*** **-value**
		**Ave. (95% CI)**	**Ave. (95% CI)**	
Day 0	%OxHSA	40.2 (38.9–41.4)	42.7 (40.8–44.5)	*0.027
	CRTAC1 (ng/mL)	171.4 (14.4–195.4)	216.6 (175.0–258.3)	0.061
Day 1	%OxHSA	39.0 (37.8–40.1)	41.6 (39.9–43.3)	*0.013
	CRTAC1 (ng/mL)	246.5 (208.5–284.6)	316.3 (250.2–382.5)	0.068
Day 7	%OxHSA	39.1 (38.0–40.3)	41.7 (40.2–43.1)	**0.007
	CRTAC1 (ng/mL)	246.7 (217.1–276.4)	328.2 (271.6–384.7)	*0.013
Day 1–7	Δ OxHSA (%)	−1.1 (−2.0 to −0.2)	−1.0 (−2.5 to −0.6)	0.870
	Δ CRTAC1 (ng/mL)	77.0 (52.6–101.5)	111.5 (56.0–167.0)	0.251

*OxHSA, oxidized albumin; CRTAC1, cartilage acidic protein-1*.

* *p < 0.05*;

** *p < 0.01*.

## Discussion

In the present study, we provided evidence of the utility of two biomarkers, %OxHSA and CRTAC1, for predicting the outcomes of AIS patients. The levels of %OxHSA in patients with poor outcomes were much higher than those of patients with better outcomes. This suggests that markedly more ROS are generated in the ischemic brain of patients with poor outcomes than in those of patients with better outcomes, and may imply that %OxHSA was relatively proportional to the amount of ROS generated. However, further research will be needed because the increase in OxHSA during ischemic stroke may be related to other physiological stress.

A study by Khatri et al. found that administering albumin within 2 h of ischemia onset improved outcomes at 3 months (30), which suggests that albumin plays a role in maintaining the whole-body reductive conditions and removes ROS by self-oxidation. The results of Khatri et al.'s study also imply that %OxHSA is not only a viable biomarker, but also an albumin treatment indicator.

To the best of our knowledge, Rael et al.'s report is the only study to date to have demonstrated the relationship between %OxHSA in blood and the outcomes of AIS patients (31). Their study revealed a slight negative correlation between mRS and %OxHSA at discharge (*r* = −0.17, *p* = 0.08), where patients with higher %OxHSA showed relatively better outcomes. Since this result was contrary to our own, we investigated whether the discordant results may have arisen from differences in the criteria used for group assignment (the mRS cutoff score was 3 in Rael et al.'s study, but 2 in our study), or from the timing of determining mRS scores (values at discharge from the hospital were employed in Rael et al.'s study, whereas those at 3 months after discharge were used in our study). We thus reassigned patients in our study into two groups according to Rael's criteria, and repeated the comparison. We found that %OxHSA in the high-mRS group (≥3 at discharge) was significantly elevated compared to that in the low-mRS group (<3 at discharge) (*p* < 0.05); thus, we determined that our conclusion was not strongly affected by the cutoff of mRS (2 or 3) or the timing of determining mRS scores (at discharge or at 3 months after discharge). We supposed that another reason for the differences in the findings of these two studies may be the difference in blood sample collection. Albumin is susceptible to an oxidant atmosphere, and is easily oxidized after blood sampling if the plasma is not kept in an acidic state. However, the efficacy, accuracy, and reliability of our %OxHSA test for AIS should be verified in a larger cohort study.

We revealed that the level of CRTAC1 in blood increases after AIS onset and that the level on day 7 after onset is strongly associated with the outcomes of AIS patients. Takase et al. revealed that overexpression of CRTAC1 in an ischemic rat model improved the neurological score significantly, with CRTAC1 inhibiting NgR1 signaling to allow regeneration of the damaged nervous system (26). Considering this positive effect of CRTAC1 on ischemic brain damage, it is plausible that high plasma CRTAC1 levels yield positive effects on the outcomes of AIS patients. However, we found the opposite result, which suggested that the amount of CRTAC1 secreted is proportional to the degree of ischemic damage. Severe ischemic damage may result in rapid and excess supply of CRTAC1 to the brain; however due to the brain's limited ability to repair itself, or insufficient supply to the damaged area, the concentration in blood remains high for a certain period of time following the ischemic injury.

It was not surprising that the level of d-dimer on admission was elevated in the high-mRS group. In the study of 2,479 patients, Zhang et al. revealed that higher d-dimer levels within 24 h after stroke onset were associated with poor functional outcome at 90 days (32). Since the correlation among the three markers was weak, i.e., they have complementary relationships, we created a combined index involving all three markers, which increased the accuracy of predicting the outcomes of AIS patients (*p* = 0.0004).

To apply these markers for the prediction of the outcomes of patients following AIS in clinical practice, several issues need to be overcome. First, since the number of enrolled patients was low in this study, it is necessary to verify the efficacy of these biomarkers in a large number of AIS patients as well as healthy persons. Second, an interventional trial, such as increasing the amount of edaravone administration to high-risk patients should be conducted. Third, CRTAC1 splice variants CRTAC-1A and−1B should ideally be distinguished, as only CRTAC1-B is thought to have NgR1 inhibitory activity, and a more selective assay may increase the sensitivity and specificity of the test.

## Conclusions

We provided insight into predicting the outcomes of AIS patients using two biomarkers, %OxHSA and CRTAC1. Higher %OxHSA and CRTAC1 are associated with worse outcomes. An index that combines %OxHSA, CRTAC1, and d-dimer can accurately predict the outcomes of AIS patients.

## Data Availability Statement

The original contributions presented in the study are included in the article/Supplementary Material, further inquiries can be directed to the corresponding author.

## Ethics Statement

The studies involving human participants were reviewed and approved by Institutional Review Board of National Hospital Organization Kyushu Medical Center (IRB registration number, 16C132) Institutional Review Board of LSI Medience Corporation (MS/Shimura 16-22). The patients/participants provided their written informed consent to participate in this study.

## Author Contributions

TK designed this work and revised the manuscript. KT provided the conception of the work and wrote the manuscript. CH did data analysis and interpretation. TM, KM, JJ, and MY collected and analyzed the patent information. YO approved final version to be published. All authors contributed to the article and approved the submitted version.

## Funding

This work was supported by LSI Medience Corporation.

## Conflict of Interest

KT and CH are employed by LSI Medience Corporation, which provides the commercially available %OxHSA and CRTAC1 tests. KT is also employed by Kyushu Pro Search Limited Liability Partnership. The authors declare that this study received funding from LSI Medience Corporation. The funder had the following involvement in the study: the study design, data collection and analysis, decision to publish, and preparation of the manuscript. The remaining authors declare that the research was conducted in the absence of any commercial or financial relationships that could be construed as a potential conflict of interest.

## Publisher's Note

All claims expressed in this article are solely those of the authors and do not necessarily represent those of their affiliated organizations, or those of the publisher, the editors and the reviewers. Any product that may be evaluated in this article, or claim that may be made by its manufacturer, is not guaranteed or endorsed by the publisher.

## Abbreviations

%OxHSA: Ratio of oxidized albumin to total albumin
8-OHdG: 8-hydroxy-2'-deoxyguanosine
AIS: acute ischemic stroke
CRTAC1: cartilage acidic protein-1
LC-MS: liquid chromatography–mass spectrometry
mRS: modified Rankin Scale
NgR1: nogo receptor-1
OxHSA: oxidized human serum albumin
PCA: principal component analysis
ROS: reactive oxygen species.
