# Supplementary material for: Oxidized Albumin and Cartilage Acidic Protein-1 as Blood Biomarkers to Predict Ischemic Stroke Outcomes
Source: Front Neurol. 2021 Nov 30;12:686555. doi: 10.3389/fneur.2021.686555 (PMC8670551; doi:10.3389/fneur.2021.686555)
Supplement: Supplementary file 2 [file Table_2.docx]

**Supporting Information**

**Oxidized albumin and cartilage acidic protein-1 as blood biomarkers to predict ischemic stroke outcomes**

Table of Contents

1. Supplemental Tables ................................................
   1. Supplementary Table I: Stability of oxidized albumin
   2. Supplementary Table II: Method validation
   3. Supplementary Table III: PCA Analysis Scores of first and second components
   4. Supplementary Table IV: PCA Analysis loadings of first and second components
2. Supplemental Figures................................................
   1. Supplementary Figure I: Typical transition of %OxHSA during admission
   2. Supplementary Figure II: Correlation between %OxHSA, CRTC1, and d-dimer
3. Supplemental Data ................................................
   1. AIS_Study_by KMC_LSIM_20210202_Final.xlsx

**1.1. Supplementary Table I: Stability of oxidized albumin**

| Condition | Number | Criterion | Result | |
| --- | --- | --- | --- | --- |
| at room temp., 2 hours after sampling, before centrifugation | 3 donors  (n = 1) | RE ≦ 5% | RE = 0.4 - 1.1% | Accepted |
| at room temp., 4 hours after sampling, before centrifugation | 3 donors  (n = 1) | RE ≦5 % | RE = 0.4 - 1.0% | Accepted |
| at room temp., 4 hours after sampling, before centrifugation | 3 donors  (n = 1) | RE ≦ 5% | RE = 0.6 - 4.6% | Accepted |
| at 4℃, 1 day after sampling, before centrifugation | 3 donors  (n = 1) | RE ≦ 5% | RE = 0.3 - 3.2% | Accepted |
| at 4℃, 3 day after sampling, before centrifugation | 3 donors  (n = 1) | RE ≦ 5% | RE = 1.0 - 2.5% | Accepted |
| at 4℃, 10 day after sampling, before centrifugation | 3 donors  (n = 1) | RE ≦ 5% | RE = 2.6 - 4.7% | Accepted |
| at -20℃, 1 month after centrifugation | 3 donors  (n = 1) | RE ≦ 5% | RE = 1.5 - 28.0% | Not accepted |
| at -80℃, 1 month after centrifugation | 3 donors  (n = 1) | RE ≦ 5% | RE = 1.4 - 2.2% | Accepted |
| at -20℃, 3 months after centrifugation | 3 donors  (n = 1) | RE ≦ 5% | RE = 32.0 - 59.9% | Not accepted |
| at -0℃, 3 months after centrifugation | 3 donors  (n = 1) | RE ≦ 5% | RE = 1.8 - 2.7% | Accepted |

**1.2. Supplementary Table II: Method validation**

| Validation | Method | Criterion | Result | |
| --- | --- | --- | --- | --- |
| Freeze-thaw stability | n=1 of 2 specimens  5 times freeze-thaw repetitions  at -20℃ and -80℃ | RE ≦ 5% | RE: - 4.1 to 2.9% | Accepted |
| Intraday reproducibility | n=6 of 3 specimens | SD ≦ 1% | SD: 0.15%, 0.48%, 0.40% | Accepted |
| Between-day reproducibility | n=1 of 3 specimens for 3 days | SD ≦ 1% | SD: 0.13%, 0.13%, 0.38% | Accepted |
| Dilution reproducibility | n=1 of 3 specimens  comparison of non-dilution and 4-fold dilution | RE ≦ 5% | RE: 1.3 to 3.6% | Accepted |
| Short period stability | n=1 of 3 specimens at room temp for 4h or 24h | RE ≦ 5% | RE: - 4.0 to - 1.1% | Accepted |

**1.3. Supplementary Table III: PCA Analysis Scores of first and second components**

| Patients | Class | PC1 t[1] | PC2 t[2] |
| --- | --- | --- | --- |
| OA0015 | Low-mRS | 0.688377 | -0.73029 |
| OA0027 | Low-mRS | 0.687921 | 0.836511 |
| OA0030 | Low-mRS | 0.45648 | 0.437594 |
| OA0031 | Low-mRS | -1.66094 | -1.68554 |
| OA0032 | Low-mRS | 5.25621 | 3.97185 |
| OA0038 | Low-mRS | -0.84075 | -1.17297 |
| OA0041 | Low-mRS | 0.255577 | -1.27126 |
| OA0043 | Low-mRS | -6.68888 | -0.66613 |
| OA0051 | Low-mRS | -0.39498 | 0.066168 |
| OA0055 | Low-mRS | -0.12722 | -2.6062 |
| OA0067 | Low-mRS | 2.34655 | -0.14451 |
| OA0076 | Low-mRS | -0.74243 | -1.90209 |
| OA0001 | Low-mRS | 1.14666 | -2.28328 |
| OA0008 | Low-mRS | -1.1915 | 0.086715 |
| OA0020 | Low-mRS | 0.038623 | 1.43912 |
| OA0024 | Low-mRS | 0.195902 | -2.13099 |
| OA0040 | Low-mRS | 2.69934 | 2.7597 |
| OA0044 | Low-mRS | -2.46116 | 0.452029 |
| OA0052 | Low-mRS | 0.998361 | 5.62802 |
| OA0056 | Low-mRS | -0.67675 | -0.14112 |
| OA0072 | Low-mRS | 0.574782 | -0.10547 |
| OA0077 | Low-mRS | 5.30959 | -0.57652 |
| OA0022 | Low-mRS | -0.27846 | -1.50795 |
| OA0068 | Low-mRS | -0.43398 | -1.56069 |
| OA0003 | Low-mRS | -3.26739 | 3.23771 |
| OA0012 | Low-mRS | 0.068139 | -3.00718 |
| OA0054 | Low-mRS | -2.06436 | 0.302623 |
| OA0007 | Low-mRS | -0.25942 | -1.25874 |
| OA0009 | Low-mRS | 2.62648 | 0.448866 |
| OA0010 | Low-mRS | 1.67389 | -3.42142 |
| OA0013 | Low-mRS | 5.13372 | 1.91081 |
| OA0016 | Low-mRS | -1.80813 | -1.18432 |
| OA0017 | Low-mRS | 4.30652 | -1.29571 |
| OA0018 | Low-mRS | 2.14368 | -2.95412 |
| OA0021 | Low-mRS | 0.431578 | -1.04494 |
| OA0026 | Low-mRS | -2.46736 | -0.74829 |
| OA0053 | Low-mRS | 2.47698 | -1.60742 |
| OA0059 | Low-mRS | -2.93857 | 0.302173 |
| OA0060 | Low-mRS | 0.7629 | 0.938053 |
| OA0063 | Low-mRS | 0.105271 | -1.17916 |
| OA0079 | Low-mRS | -0.14642 | 0.094471 |
| OA0081 | Low-mRS | -2.52822 | -0.17704 |
| OA0048 | Low-mRS | 2.68472 | -2.20037 |
| OA0062 | Low-mRS | 2.86786 | 0.386836 |
| OA0046 | High-mRS | 0.618929 | -0.93928 |
| OA0080 | High-mRS | -0.83266 | -0.54678 |
| OA0019 | High-mRS | 0.663243 | 3.35003 |
| OA0071 | High-mRS | -0.84678 | 0.200045 |
| OA0002 | High-mRS | 0.587797 | -2.36417 |
| OA0078 | High-mRS | 3.92217 | 2.74119 |
| OA0049 | High-mRS | -2.10003 | -1.74219 |
| OA0005 | High-mRS | -2.21911 | 6.40032 |
| OA0023 | High-mRS | -2.19313 | -0.46126 |
| OA0029 | High-mRS | -1.35698 | -1.31322 |
| OA0069 | High-mRS | -0.69262 | 1.10478 |
| OA0070 | High-mRS | -3.25847 | 1.21606 |
| OA0014 | High-mRS | 1.26429 | -1.80549 |
| OA0036 | High-mRS | -3.07183 | -1.05093 |
| OA0037 | High-mRS | -0.41433 | 1.93828 |
| OA0073 | High-mRS | 6.12981 | 2.0681 |
| OA0045 | High-mRS | -0.6697 | -1.63394 |
| OA0004 | High-mRS | -2.82937 | 4.26311 |
| OA0042 | High-mRS | -0.04944 | -0.45198 |
| OA0057 | High-mRS | -0.34127 | 3.11053 |
| OA0058 | High-mRS | -0.18115 | -0.6161 |
| OA0065 | High-mRS | -1.51622 | -1.8605 |
| OA0066 | High-mRS | 1.52581 | -1.12932 |
| OA0074 | High-mRS | 2.92037 | 1.09275 |
| OA0006 | High-mRS | -6.71517 | 1.85121 |
| OA0075 | High-mRS | -3.66 | 1.82549 |

**1.4. Supplementary Table IV: PCA Analysis loadings of first and second components**

| Var ID (Primary) | PC1 p[1] | PC2 p[2] |
| --- | --- | --- |
| Systolic arterial pressure | 0.112051 | -0.00933 |
| Diastolic pressure | 0.135413 | 0.053304 |
| White blood cells | 0.013586 | 0.265519 |
| Red blood cells | 0.279029 | 0.206179 |
| Hematocrit | 0.2298 | 0.195982 |
| Hemoglobin | 0.27796 | 0.191035 |
| Platelet | 0.150689 | 0.13513 |
| AST | 0.015399 | 0.24297 |
| ALT | 0.128984 | 0.212842 |
| LDH | -0.02301 | 0.183603 |
| ALP | -0.00937 | 0.145374 |
| γ-GTP | 0.125093 | 0.099866 |
| T-Bil | 0.037174 | 0.115089 |
| Total cholesterol | 0.273135 | -0.02439 |
| LDL cholesterol | 0.237357 | 0.023518 |
| HDL cholesterol | 0.008854 | -0.11592 |
| TG | 0.159744 | 0.010219 |
| TP | 0.075319 | -0.04762 |
| Albumin | 0.15164 | -0.17648 |
| CPK | -0.02947 | 0.078314 |
| BUN | -0.23778 | 0.013562 |
| Cr | -0.1818 | 0.032653 |
| eGFR | 0.283651 | -0.05396 |
| Urinary acid | -0.05761 | 0.107537 |
| FBS | 0.106702 | 0.269685 |
| BS | 0.149039 | 0.207428 |
| HbA1c | 0.181681 | 0.225847 |
| CRP | -0.07951 | 0.242346 |
| PT | -0.11447 | 0.127846 |
| APTT | -0.09405 | 0.011756 |
| Fibrinogen | -0.00554 | 0.224026 |
| D-dimer | -0.19023 | 0.068936 |
| OxHSA (day0) | -0.19987 | 0.296412 |
| CRTAC1 (day0) | -0.21545 | 0.112537 |
| OxHSA (day1) | -0.23066 | 0.263784 |
| CRTAC1 (day1) | -0.14646 | 0.102168 |
| OxHSA (day7) | -0.17248 | 0.208716 |
| CRTAC1 (day7) | -0.14987 | 0.13234 |

**2.1. Supplementary Figure I: Typical transition of %OxHSA during admission**

**
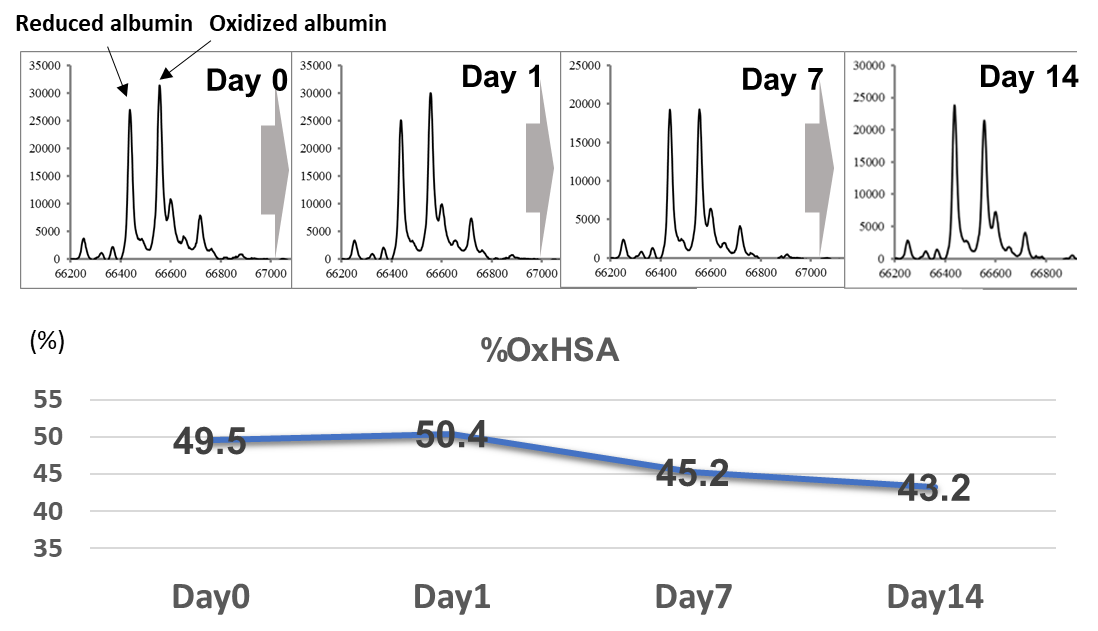
**

**2.2. Supplementary Figure II: Correlation between %OxHSA, CRTC1, and d-dimer**

**
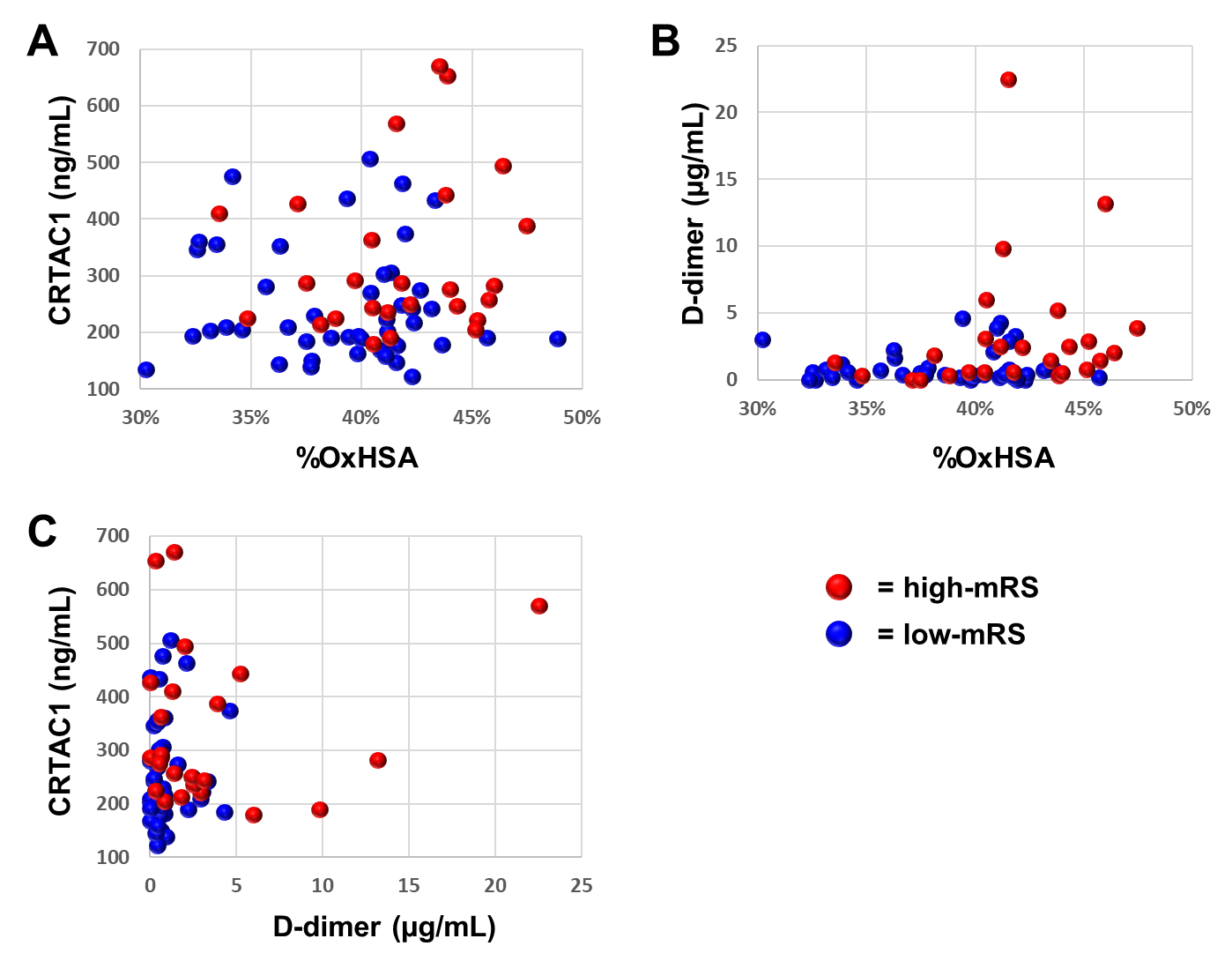
**
